# Physical, Tensile, and Microstructural Properties of Jute/False Banana Fiber-Reinforced Unsaturated Polyester Resin Composites

**DOI:** 10.3390/polym18172080

**Published:** 2026-08-27

**Authors:** Mert Yildirim

**Affiliations:** 1 Department of Industrial Engineering, Istanbul Gelisim University, Istanbul 34310, Türkiye; meryildirim@gelisim.edu.tr; 2 New Generation Entrepreneurship and Innovation Application and Research Center, Istanbul Gelisim University, Istanbul 34310, Türkiye

**Keywords:** jute fiber, false banana fiber, natural fiber-reinforced composites, unsaturated polyester resin, vacuum infusion, water absorption, tensile properties, microstructural analysis

## Abstract

In this study, composites reinforced with jute fiber, false banana fiber, and hybrid jute/false banana fibers were fabricated using unsaturated polyester resin (UPR) as the matrix material via the vacuum infusion method. To examine the effects of fiber type and hybridization on density, water absorption, tensile behavior, and microstructural features, all composite samples were prepared with a fixed total fiber content of 30 wt.% and a UPR matrix content of 70 wt.%. Among the fabricated composites, the false banana fiber-reinforced polymer composite (FBFRPC) exhibited the lowest density, with a value of 1.16 g/cm^3^, whereas the jute fiber-reinforced polymer composite (JFRPC) showed the highest density of 1.22 g/cm^3^. Water absorption increased gradually with immersion time in all composite groups. After 72 h of immersion, the lowest water absorption was recorded for the JFRPC, at 5.30%, while the highest value was obtained for the FBFRPC at 6.20%. Scanning electron microscopy (SEM) analysis revealed that fiber type caused noticeable differences in fiber dispersion, resin impregnation, fiber pull-out behavior, and fiber–matrix interfacial bonding. The JFRPC demonstrated the highest tensile performance, reaching a maximum tensile stress of 51.20 MPa and a Young’s modulus of 885.60 MPa. In contrast, the FBFRPC showed the lowest tensile strength but achieved the highest strain value of 7.40%, indicating greater deformation capability before fracture. Compared with the individual jute and false banana fiber composites, the hybrid jute/false banana fiber-reinforced polymer composite (JFBFRPC) provided a balanced combination of density, water absorption, microstructural characteristics, and tensile properties. Overall, the findings indicate that hybridization is an effective approach for developing lightweight unsaturated polyester composites reinforced with renewable lignocellulosic fibers for potential non-load-bearing and semi-structural applications.

## 1. Introduction

Synthetic fibers are extensively used as reinforcing materials in polymer-based composites because of their high strength, rigidity, durability, and consistent engineering performance [1]. Glass, carbon, and Kevlar/aramid fibers are among the most widely preferred synthetic reinforcements for structural and semi-structural composite applications [2]. Nevertheless, the manufacture of these fibers generally requires high energy consumption and depends mainly on non-renewable petroleum-derived or petrochemical resources [3]. Moreover, issues such as poor biodegradability, relatively high processing costs, difficulties in end-of-life disposal, and environmental concerns have increased the need to reconsider the sustainability of conventional fiber-reinforced polymer composites.

In recent years, natural fiber-reinforced polymer composites (NFRPCs) have gained considerable interest in academic studies and industrial fields. In comparison with many conventional synthetic reinforcements, natural fibers offer several potential advantages, including low density, renewable origin, and, depending on the material system and end-of-life conditions, potential environmental benefits [4,5,6]. Furthermore, natural fibers can lessen dependence on non-renewable and energy-intensive materials, improve environmental performance, support circular economy practices, and contribute to the attainment of the Sustainable Development Goals (SDGs) of the United Nations [7,8]. However, the environmental performance of a specific composite system depends on its complete life cycle, including matrix production, fiber processing, manufacturing, transportation, use, and end-of-life scenarios.

Recent studies on fiber-reinforced composite laminates have demonstrated that laminate architecture, stacking sequence, fiber orientation, and the relative positioning of reinforcement layers can significantly influence mechanical performance, load transfer, and failure behavior [9]. These parameters are particularly important in hybrid composite systems, where the arrangement of different reinforcement types can be tailored to achieve a desired balance of physical and mechanical properties [10]. NFRPCs have also attracted increasing attention for lightweight engineering applications, including automotive, construction, infrastructure, and other non-load-bearing or semi-structural components [6]. Recent review studies have further highlighted the potential of natural and hybrid fiber-reinforced polymer composites for lightweight material systems incorporating renewable reinforcements, while identifying moisture sensitivity, fiber–matrix compatibility, processing variability, interfacial bonding, and environmental durability as key challenges for broader engineering implementation [6,10].

Natural fibers are commonly classified by their origin as plant-based, animal-based, or mineral-based fibers [11]. Among these categories, plant-derived lignocellulosic fibers are the most frequently used in polymer composite applications due to their wide availability, low density, renewable nature, acceptable mechanical performance, and relatively low cost [12]. Fibers such as jute, flax, hemp, sisal, coir, cotton, bamboo, banana, and kenaf have been widely examined as reinforcing materials in both thermoset and thermoplastic polymer matrices [8]. Among these fibers, jute and false banana fibers, like most natural cellulosic fibers, are mainly composed of cellulose, hemicellulose, lignin, and other minor constituents [13,14]. However, the overall performance of natural fiber-reinforced composites depends on various parameters, including fiber type, fiber loading, fiber orientation, fiber morphology, moisture uptake, processing conditions, and fiber–matrix interfacial bonding [15].

Jute is a lignocellulosic bast fiber mainly obtained from *Corchorus capsularis* and *Corchorus olitorius*. It is grown extensively in South Asia, particularly in Bangladesh and India, and is regarded as one of the most commercially significant natural fibers after cotton [16]. Due to its characteristic color and glossy appearance, jute is commonly referred to as the “golden fiber.” Jute fibers offer low density, high aspect ratio, a favorable strength-to-weight balance, acceptable tensile properties, and good thermal and acoustic insulation performance [17]. Therefore, jute fiber-reinforced polymer composites (JFRPCs) have attracted attention for applications such as automotive interior components, furniture panels, packaging materials, building panels, and lightweight semi-structural products [18,19].

Earlier research has highlighted the suitability of jute fibers as reinforcing materials in polymer composites. Studies on jute fiber-reinforced polymer composites (JFRPCs) have reported that adding jute fibers can improve mechanical performance; however, moisture sensitivity and insufficient fiber–matrix adhesion remain important challenges [20,21,22]. Research on jute-based hybrid composites has also shown that hybridization, fiber arrangement, stacking sequence, and reinforcement architecture can markedly influence tensile behavior, damage development, and water absorption characteristics. These results indicate that jute fibers have considerable potential for polymer composite applications, although their performance is strongly dependent on interfacial bonding, processing quality, and resistance to moisture uptake.

False banana, also known as enset (*Ensete ventricosum*), is a large herbaceous perennial species from the Musaceae family. It is predominantly grown in Ethiopia and is considered an important multipurpose crop due to its nutritional, fibrous, and socioeconomic contributions [23,24]. Although it resembles the common banana plant in appearance, false banana is mainly cultivated for its pseudostem, corm, and leaf sheath rather than its fruit.

Beyond its nutritional importance, enset plays a significant role in food security and rural livelihoods in Ethiopia, where it serves as a staple crop for millions of people. Its tolerance to drought and its ability to remain in the field until harvesting is required further enhance its value as a resilient crop. In addition, the utilization of enset-derived biomass in high-value applications offers opportunities for more sustainable and bio-based value chains. The fibrous tissues obtained from the pseudostem and leaf sheath provide a valuable source of natural fiber, enabling agricultural residues and processing by-products to be converted into value-added reinforcement materials.

In recent years, false banana fiber has attracted increasing attention as a renewable lignocellulosic reinforcement for polymer composites [25,26,27,28]. Its lignocellulosic nature, relatively high cellulose content, low density, renewable origin, and favorable mechanical properties make it a promising reinforcement material for composite applications. In general, natural fibers with high cellulose content can enhance stiffness and tensile load-bearing capacity; however, their reinforcing efficiency is strongly influenced by fiber morphology, chemical composition, extraction process, structural imperfections, moisture content, and interfacial compatibility with the polymer matrix. Like many lignocellulosic fibers, false banana fiber is hydrophilic, which can increase moisture uptake and weaken interfacial bonding with hydrophobic polymer matrices, thereby limiting the overall performance of the resulting composites.

The hybridization of two or more natural fibers is a useful strategy for enhancing or balancing the properties of natural fiber-reinforced composites [29]. By combining fibers with different morphologies, chemical structures, and mechanical characteristics, hybrid composites can provide improved strength, stiffness, density, moisture response, and damage resistance compared with single-fiber systems. In this context, combining widely used jute fiber with relatively less-studied false banana fiber may offer a promising approach for producing lightweight composites with balanced physical and mechanical performance while incorporating renewable natural reinforcements.

Recent studies have emphasized that the performance of natural fiber-reinforced polymer composites depends not only on the intrinsic properties of the reinforcing fibers but also on fiber morphology and architecture, fiber–matrix compatibility, processing conditions, moisture sensitivity, and interfacial bonding [6,30]. These parameters directly affect stress transfer, damage development, dimensional stability, and the overall suitability of natural fiber composites for engineering applications. In this context, false banana (*Ensete ventricosum*) fiber has received increasing attention as an underutilized lignocellulosic reinforcement because of its renewable origin, relatively low density, and potential for value-added utilization in polymer composites [24]. Nevertheless, compared with more established natural fibers such as jute, flax, and conventional banana fiber, the available literature on false banana fiber-reinforced polymer composites (FBFRPCs) remains comparatively limited, particularly with regard to hybrid reinforcement systems, moisture response, and fiber–matrix interactions.

Previous studies have reported that the incorporation of natural fibers or natural fillers can modify and, under appropriate conditions, improve the mechanical behavior of polymer composites [30,31,32,33,34]. Although JFRPCs have been extensively investigated, considerably less attention has been given to false banana (*Ensete ventricosum*) fiber and, particularly, to its hybridization with jute fiber in an unsaturated polyester resin matrix. Previous studies have generally focused on individual natural fiber systems, different reinforcement contents, or different processing conditions, which makes direct comparison of the respective contributions of jute and false banana fibers difficult. Therefore, the significance of the present study lies in the systematic comparison of jute fiber-reinforced polymer composite (JFRPC), false banana fiber-reinforced polymer composite (FBFRPC), and hybrid jute/false banana fiber-reinforced polymer composite (JFBFRPC), all fabricated under identical processing conditions and with a constant total fiber content of 30 wt.%. This controlled experimental design enables the effects of fiber type and hybridization on density, water absorption, tensile behavior, and fracture morphology to be evaluated while minimizing variations associated with differences in total reinforcement content. Furthermore, the study provides directly comparable data for the relatively underexplored jute/false banana hybrid system and contributes to assessing the potential of false banana fiber as a complementary natural reinforcement for lightweight unsaturated polyester composites.

## 2. Materials and Methods

### 2.1. Materials

Jute fibers sourced from the Faridpur region of Bangladesh and false banana (*Ensete ventricosum*) fibers sourced from the southern region of Ethiopia were used as natural reinforcement materials. A commercial cobalt-accelerated unsaturated polyester resin (UPR), supplied by Boytek (Istanbul, Türkiye), was used as the polymer matrix. Methyl ethyl ketone peroxide (MEKP), procured from Merck (Darmstadt, Germany), was added at 1 wt.% as the curing initiator.

### 2.2. Formulation of Composites

Table 1 presents the weight fractions of the composite formulations prepared in this study.

Three composite formulations were prepared while maintaining a constant unsaturated polyester resin (UPR) content of 70 wt.% and a constant total fiber content of 30 wt.%. Sample A was reinforced only with jute fiber, whereas Sample B was reinforced only with false banana fiber. Sample C was prepared as a hybrid composite containing equal weight fractions of jute and false banana fibers.

### 2.3. Fabrication of Composites

**Figure 1:**
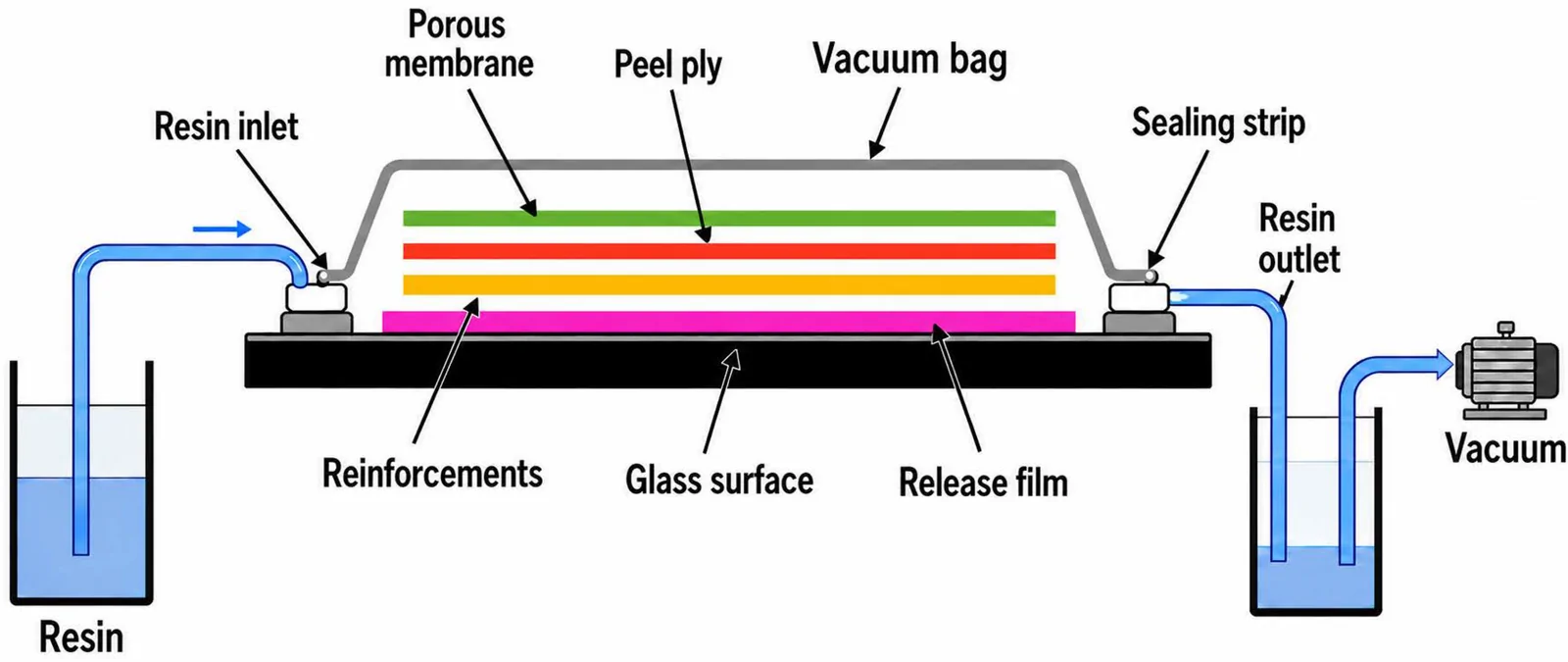
Visual representation of the composite fabrication procedure using the vacuum infusion method.

The composite specimens were produced through vacuum infusion, which was selected to ensure uniform resin distribution, effective fiber impregnation, and improved structural homogeneity throughout the composites. Figure 1 schematically illustrates the procedure used to fabricate the composites.

Before fabrication, the glass mold was first wiped with a damp cloth and then thoroughly dried using a clean, dry cloth. Masking tape was applied to define the fabrication area, ensuring that the bordered region was at least 50 mm larger than the edge dimensions of the prepared natural fiber layers. To facilitate demolding, three layers of mold release wax were applied to the confined area using a lint-free cloth, with an interval of approximately 5 min between successive layers to allow proper surface drying.

The reinforcement layers were then arranged on the waxed mold surface according to the composite formulations described in Table 1. For Sample C, the jute fiber layer was placed first on the mold surface, followed by the false banana fiber layer, resulting in a two-layer jute/false banana stacking sequence rather than a randomly intermixed hybrid reinforcement. Each fiber type constituted 15 wt.% of the composite, resulting in a total fiber content of 30 wt.%. The fibers, with lengths of 300–600 mm, were distributed as uniformly as possible within their respective layers. Once the fiber layers had been positioned, the masking tape was removed, and vacuum sealant tape was applied around the perimeter of the prepared region.

To facilitate demolding after curing, a peel ply was laid over the fiber reinforcement. The peel ply was cut and positioned to extend approximately 20 mm beyond the fiber dimensions on three sides and 60 mm beyond the fiber dimensions on the resin inlet side. A spiral tube connected to a transparent resin feed tube was used to introduce the resin into the system. The spiral tube was wrapped with the 60 mm extended portion of the peel ply, fixed in place, and secured with masking tape to prevent unwinding.

To enhance resin flow and promote homogeneous impregnation, a flow mesh was placed over the peel ply. The flow mesh was cut to extend approximately 10 mm beyond the peel ply on three sides and 20 mm beyond the peel ply on the resin inlet side. The spiral tube was fixed to the flow mesh using masking tape. The vacuum line was installed on the side opposite the resin inlet line. Before positioning the vacuum and resin hoses onto the vacuum sealing tape, an additional layer of sealing tape was wrapped around the hose-bonding regions to minimize air leakage. The resin feed hose was then connected to the spiral tube, completing the infusion setup.

After the prepared lay-up was covered and sealed with a vacuum bag, the vacuum pump was used to remove trapped air from the system. The vacuum bag was drawn tightly against the mold surface to ensure proper consolidation of the fiber layers. The matrix system was prepared by adding 1 wt.% MEKP to the cobalt-accelerated unsaturated polyester resin. The final composite formulation consisted of 30 wt.% total fiber reinforcement and 70 wt.% UPR resin.

After vacuum stabilization, the resin inlet hose was opened to initiate resin infusion. Once the fiber layers were completely impregnated with resin, the inlet hose was folded, closed, and secured with a zip tie to prevent backflow. Following full wetting of the reinforcement, the vacuum pump was turned off, and the laminate was cured at room temperature for 48 h. Following the curing stage, the composite samples were demolded and cut to the specimen geometries and dimensions required by the relevant testing standards.

**Figure 2:**
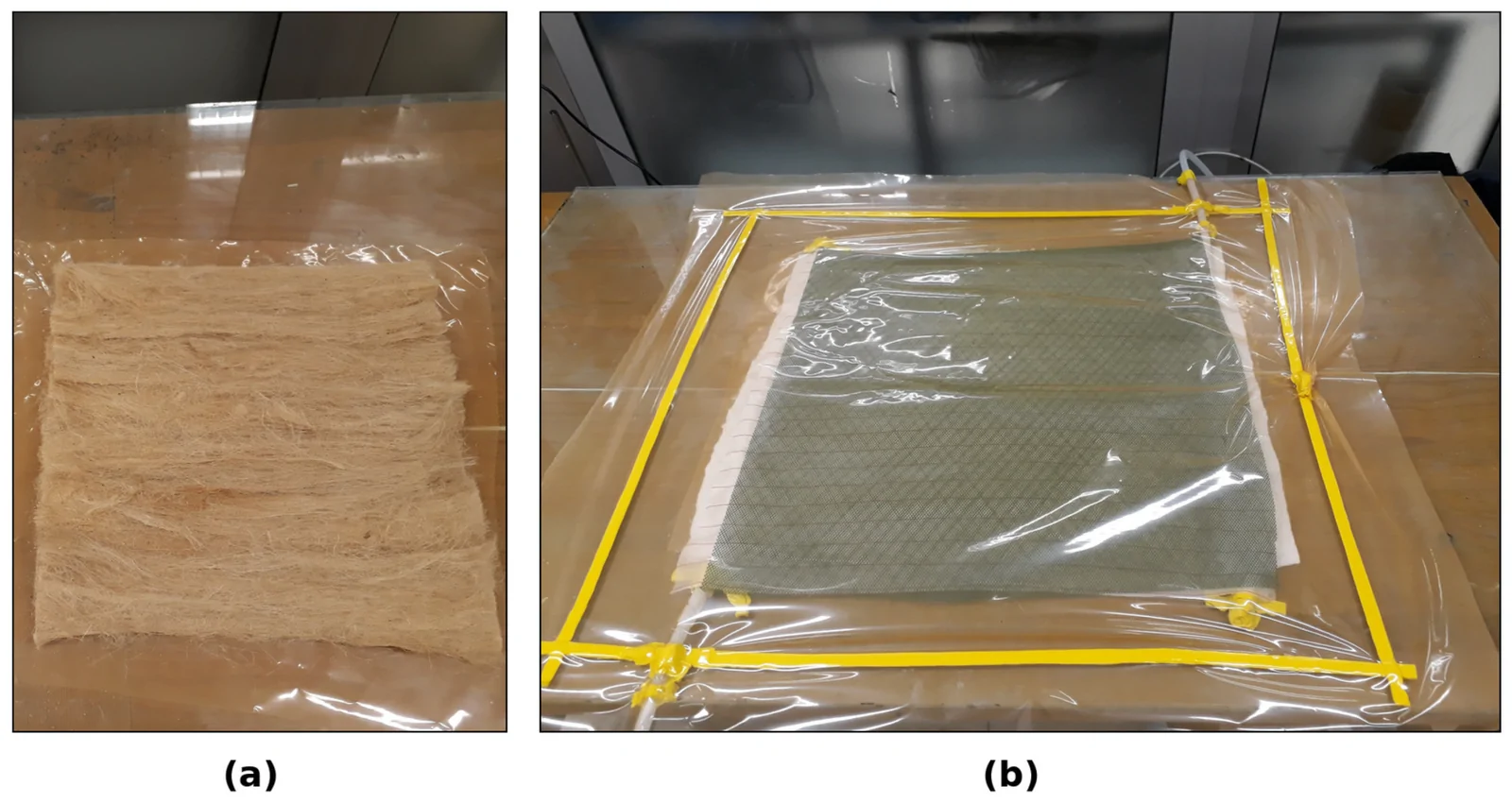
Representative photographs of the composite preparation procedure: (**a**) prepared jute fiber reinforcement layer prior to composite fabrication; (**b**) vacuum bagging and infusion setup prior to resin infusion.

Representative photographs of the jute fiber reinforcement layer and the vacuum bagging/infusion setup used during composite fabrication are shown in Figure 2.

### 2.4. Characterization

#### 2.4.1. Physical Characterization

Density and water absorption analyses were carried out to determine the physical behavior of the fiber-reinforced composite specimens. For these tests, samples with dimensions of 50 mm × 50 mm × composite thickness were prepared, with the thickness varying between 3 and 4 mm. Specimen dimensions were measured using a digital caliper and micrometer (Mitutoyo, Kawasaki, Japan). Density measurements were performed following ASTM D792 [35]. The specimens were weighed using a digital analytical balance with a readability of 0.0001 g (Mettler Toledo, Greifensee, Switzerland). Water absorption testing was conducted following ASTM D1037-2006 [36] using a temperature-controlled water bath (TERMAL, Istanbul, Türkiye) maintained at 22 ± 1 °C.

Before the test, the specimens were dried to remove residual moisture and subsequently weighed using the analytical balance to obtain their initial dry mass (Wi). Subsequently, the samples were placed vertically in the temperature-controlled water bath at 22 ± 1 °C. Water absorption was evaluated after immersion durations of 2, 24, 48, and 72 h. At each specified time point, the specimens were taken from the bath, carefully dried on the surface using absorbent paper to remove residual moisture, and immediately weighed to obtain their final mass (Wf). For density and water absorption measurements, five replicate specimens were tested for each composite group (n = 5), and the mean values were used for evaluation. The percentage of water absorption (WA) was calculated using the equation given below:

WA (%) = {[W*_f_* − W*_i_*]/W*_i_*} × 100

#### 2.4.2. Scanning Electron Microscopy (SEM) Characterization

Three representative fractured specimens from each composite group were selected after tensile testing for scanning electron microscopy (SEM) examination. The fractured surfaces of the composite test specimens were examined using a Quanta FEG 450 scanning electron microscope (FEI, Hillsboro, OR, USA) equipped with a backscattered electron detector (BSED). Before SEM observation, the specimens were mounted on SEM stubs and coated with a thin gold layer using a Quorum Q150T ES sputter coater (Quorum Technologies, Lewes, East Sussex, UK) to enhance electrical conductivity and reduce charging effects. During SEM imaging, the microscope was operated in high-vacuum mode at 6 kV, with the working distance adjusted within the range of 10.6–12.4 mm. Micrographs were captured at magnifications of 100×, 500×, and 1000×. The SEM examination was conducted as a qualitative fractographic assessment of fiber–matrix interaction and failure features rather than as a quantitative measurement of surface roughness or pore-size distribution.

#### 2.4.3. Tensile Characterization

Tensile specimens measuring 20 mm × 200 mm were fabricated in accordance with ASTM D3039 [37]. The tensile response of the specimens was determined using a Devotrans DVT GP D NN universal testing machine (Devotrans, Istanbul, Türkiye) equipped with a 10 kN load cell. All measurements were performed at room temperature in displacement-control mode. A constant crosshead speed of 2 mm/min was maintained until the specimens underwent complete fracture. Five replicate specimens were tested for each composite group (n = 5), and the results are reported as mean ± standard deviation.

**Figure 3:**
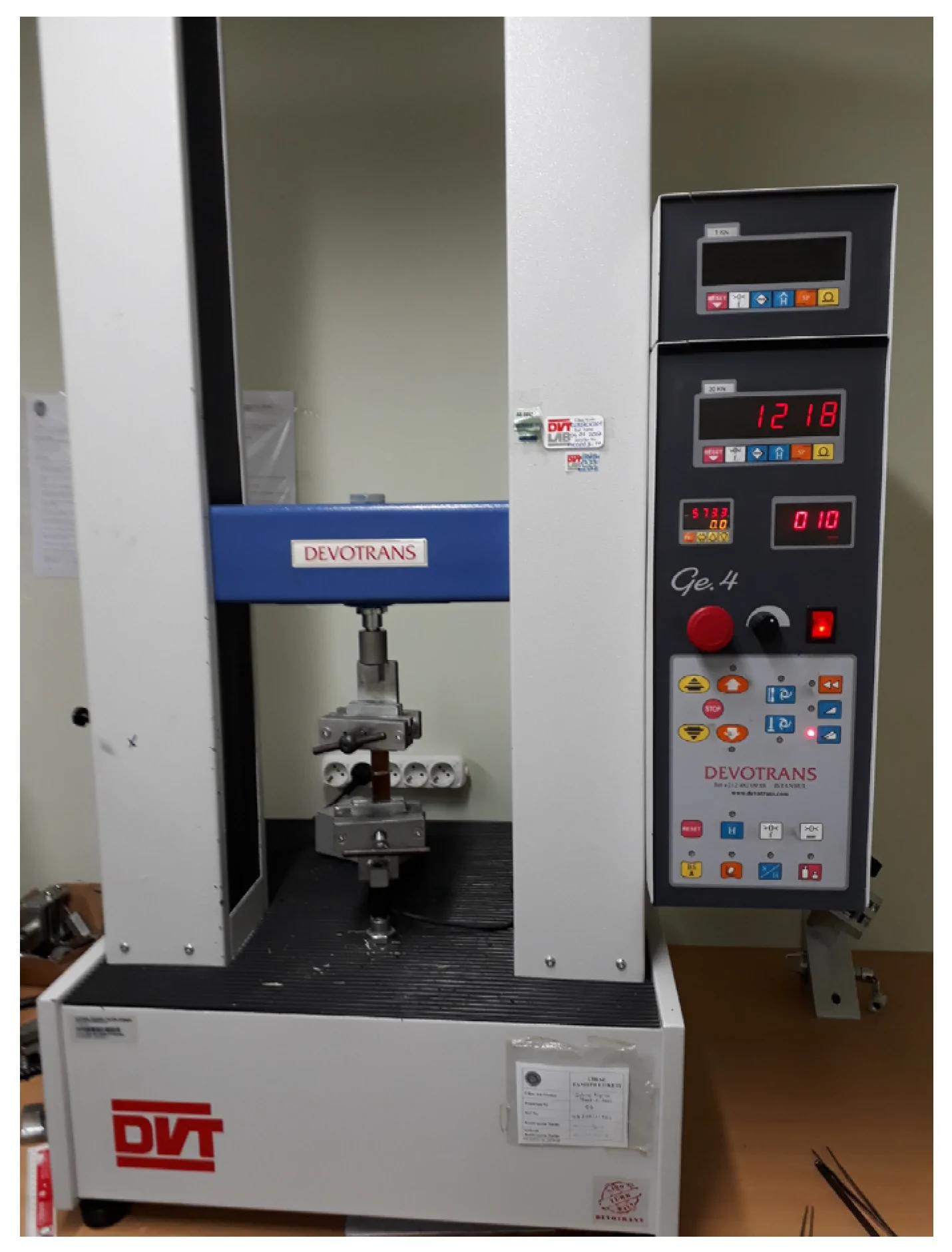
Representative photograph of the Devotrans DVT GP D NN universal testing machine used for tensile testing.

A representative photograph of the universal testing machine used for the tensile tests is shown in Figure 3.

## 3. Results and Discussion

### 3.1. Density

The density results of the composites are presented in Table 2.

Table 2 indicates that sample A had the highest density at 1.22 g/cm^3^, whereas sample B exhibited the lowest value at 1.16 g/cm^3^. Sample C showed an intermediate density of 1.19 g/cm^3^.

Since all composites were fabricated with a fixed unsaturated polyester resin (UPR) matrix content of 70 wt.% and a total fiber loading of 30 wt.%, the variations in density are mainly associated with the fiber type and the relative fiber composition within the composite system. The higher density of sample A suggests that the presence of jute fiber produced a more compact and denser composite structure. Conversely, the reduced density of sample B indicates that false banana fiber contributed to a lighter composite structure. The intermediate density observed for sample C can be attributed to the hybrid reinforcement effect, where the combined use of jute and false banana fibers resulted in a density value between those of the corresponding single-fiber composites.

These results indicate that replacing part or all of the jute fiber with false banana fiber can reduce the density of UPR-based composites while maintaining the same total fiber loading. Therefore, the sample C provides a balanced density response compared with the single-fiber composites. In general, the density results highlight the lightweight nature of the produced natural fiber-reinforced UPR composites, offering an advantage for applications that require weight-efficient material design.

The density results obtained in the present study are in agreement with earlier findings, which reported that natural fiber-reinforced polymer composites are suitable for producing lightweight structures because lignocellulosic fibers generally have lower densities than conventional synthetic reinforcements [38].

### 3.2. Water Absorption

Table 3 presents the water absorption (WA) results of the composites measured after immersion durations of 2, 24, 48, and 72 h.

Table 3 indicates that water absorption increased progressively with longer immersion periods for all composite specimens. This behavior is commonly observed in natural fiber-reinforced polymer composites (NFRPCs) because of the hydrophilic nature of lignocellulosic fibers. The hydroxyl groups present in natural fibers promote moisture uptake, while water can gradually diffuse into the composite structure through fiber-rich regions, fiber–matrix interfaces, and possible microvoids.

Sample A, reinforced solely with jute fiber, exhibited the lowest water absorption throughout the immersion period. Its water absorption increased from 1.35% after 2 h to 5.30% after 72 h. This lower moisture uptake suggests that the jute fiber-reinforced composite had relatively better resistance to water absorption than the other samples, possibly due to a more compact composite structure and improved fiber–matrix compatibility.

Sample B, reinforced solely with false banana fiber, exhibited the highest water absorption at all immersion times, increasing from 1.60% after 2 h to 6.20% after 72 h. The higher moisture uptake of this sample may be associated with the greater hydrophilicity of false banana fiber, as well as its tendency to absorb water within the unsaturated polyester resin (UPR) matrix. This finding indicates that composites reinforced with false banana fibers exhibited greater water uptake than those containing jute fibers.

Sample C exhibited intermediate water absorption, increasing from 1.45% after 2 h to 5.75% after 72 h. Its water absorption values remained between those of samples A and B at all immersion times, indicating that hybridization produced a balanced moisture absorption response. The incorporation of jute fiber into false banana fiber-reinforced composite resulted in lower water absorption in the hybrid JFBFRPC compared with the single-fiber FBFRPC.

It was also observed that the increase in water absorption was more pronounced during the early immersion period and became more gradual at longer immersion times. This trend can be attributed to the rapid initial penetration of water into accessible fiber-rich regions and interfacial pathways. As immersion time increased, the diffusion rate gradually decreased due to the progressive saturation of the composite structure.

Overall, the results indicate that the type of natural fiber significantly influenced the water absorption behavior of the composites. Sample A exhibited the most favorable moisture resistance, whereas Sample B showed the highest water absorption. Sample C exhibited intermediate behavior, suggesting that hybridization may provide an effective approach for balancing the physical properties of NFRPCs. The continuous increase in water absorption with immersion time agrees with the general behavior reported for lignocellulosic fiber-reinforced polymer composites. Moisture uptake in NFRPCs is mainly attributed to the hydrophilic nature of lignocellulosic fibers, which contain hydroxyl groups in cellulose, hemicellulose, and lignin that promote hydrogen bonding with water molecules. Previous studies have also indicated that water can penetrate NFRPCs through fiber–matrix interfaces, microvoids, cracks, and fiber-rich regions [39,40]. Therefore, the higher water absorption of the Sample B can be associated with the hydrophilic character of the fiber, possible interfacial gaps, and less uniform matrix impregnation, as also supported by the scanning electron microscopy (SEM) observations. In addition, the intermediate water absorption behavior of the hybrid composite is consistent with previous reports indicating that natural fiber hybridization can provide a balanced response by combining the characteristics of different lignocellulosic reinforcements [41].

### 3.3. Scanning Electron Microscopy (SEM)

The microstructural characteristics of the fabricated composites were investigated using scanning electron microscopy (SEM). The SEM micrographs were qualitatively evaluated in terms of fiber distribution, fiber–matrix interfacial features, matrix coverage, apparent interfacial discontinuities, exposed or fractured fibers, and fracture surface morphology.

**Figure 4:**
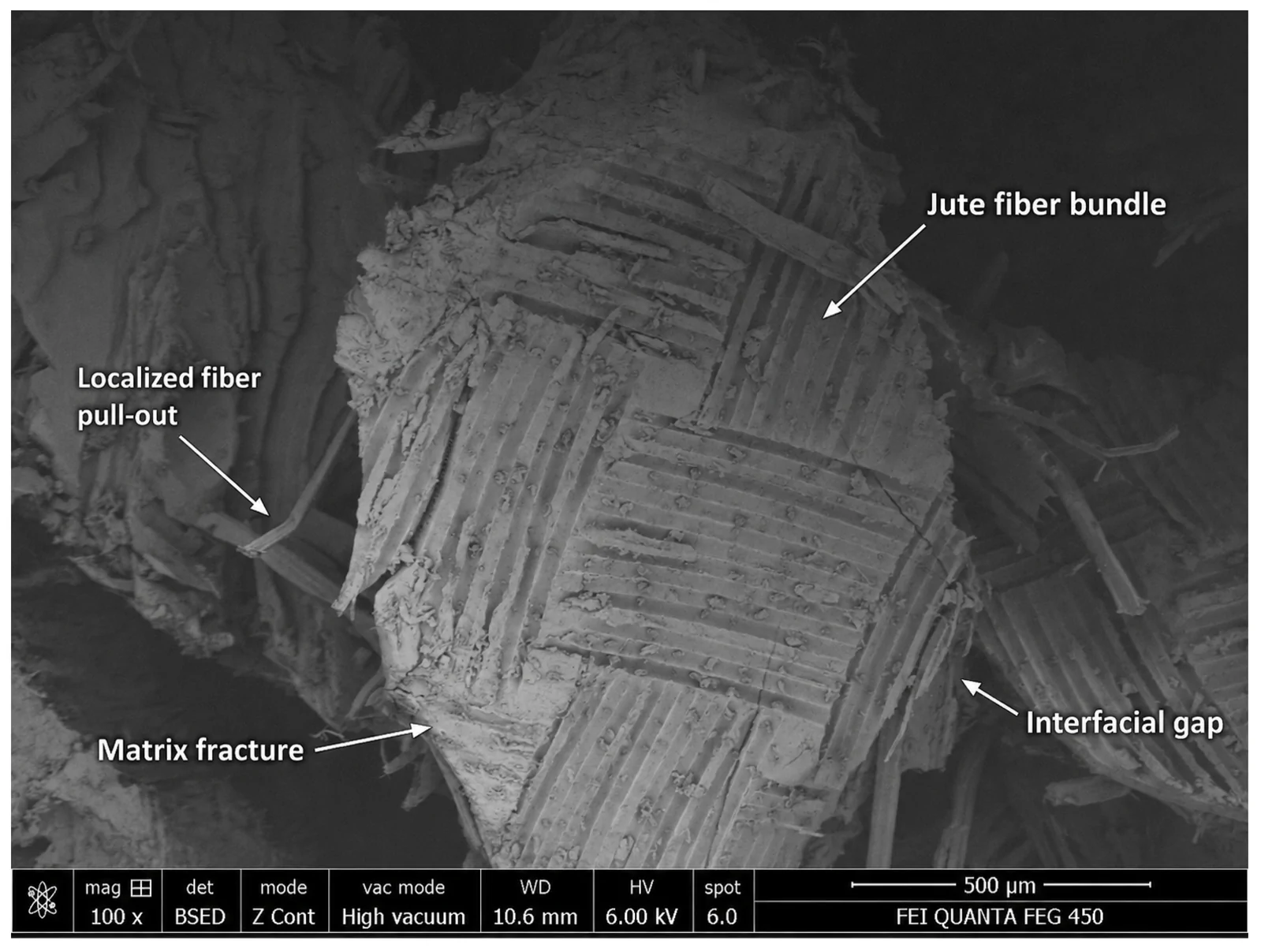
Scanning electron microscopy (SEM) micrograph of sample A containing 30 wt.% jute fiber and 70 wt.% unsaturated polyester resin (UPR). Arrows indicate representative fractographic features, including jute fiber bundles, localized fiber pull-out, fractured matrix regions, and interfacial separation/gaps.

The SEM image of specimen A, containing 30 wt.% jute fiber and 70 wt.% UPR, is presented in Figure 4.

As observed in Figure 4, distinct jute fiber bundles were embedded within the polyester matrix. The fiber bundles exhibited a relatively dense and partially oriented morphology. The matrix-covered regions surrounding the bundles are consistent with resin impregnation during composite fabrication. The principal fractographic features identified in Figure 4 include jute fiber bundles, localized fiber pull-out, fractured matrix regions, and interfacial separation/gaps.

Localized interfacial discontinuities, including interfacial separation/gaps and limited fiber pull-out regions, can also be observed. These features indicate that fiber–matrix interaction was not completely uniform throughout the composite structure. Such features may be associated with the irregular geometry of natural fibers, variations in fiber thickness, and limited resin penetration into compact fiber bundles. The textured surface of the jute fibers may promote mechanical anchoring within the polyester matrix, whereas localized interfacial discontinuities may hinder effective load transfer in certain regions.

Overall, the SEM observation indicates that sample A exhibited a typical JFRPC morphology characterized by relatively compact fiber bundles, matrix-covered regions, localized fiber pull-out, and interfacial discontinuities. These qualitative microstructural observations are consistent with the comparatively higher tensile strength and Young’s modulus obtained for sample A, although the SEM analysis was not intended to provide quantitative measurements of interfacial bonding or porosity.

**Figure 5:**
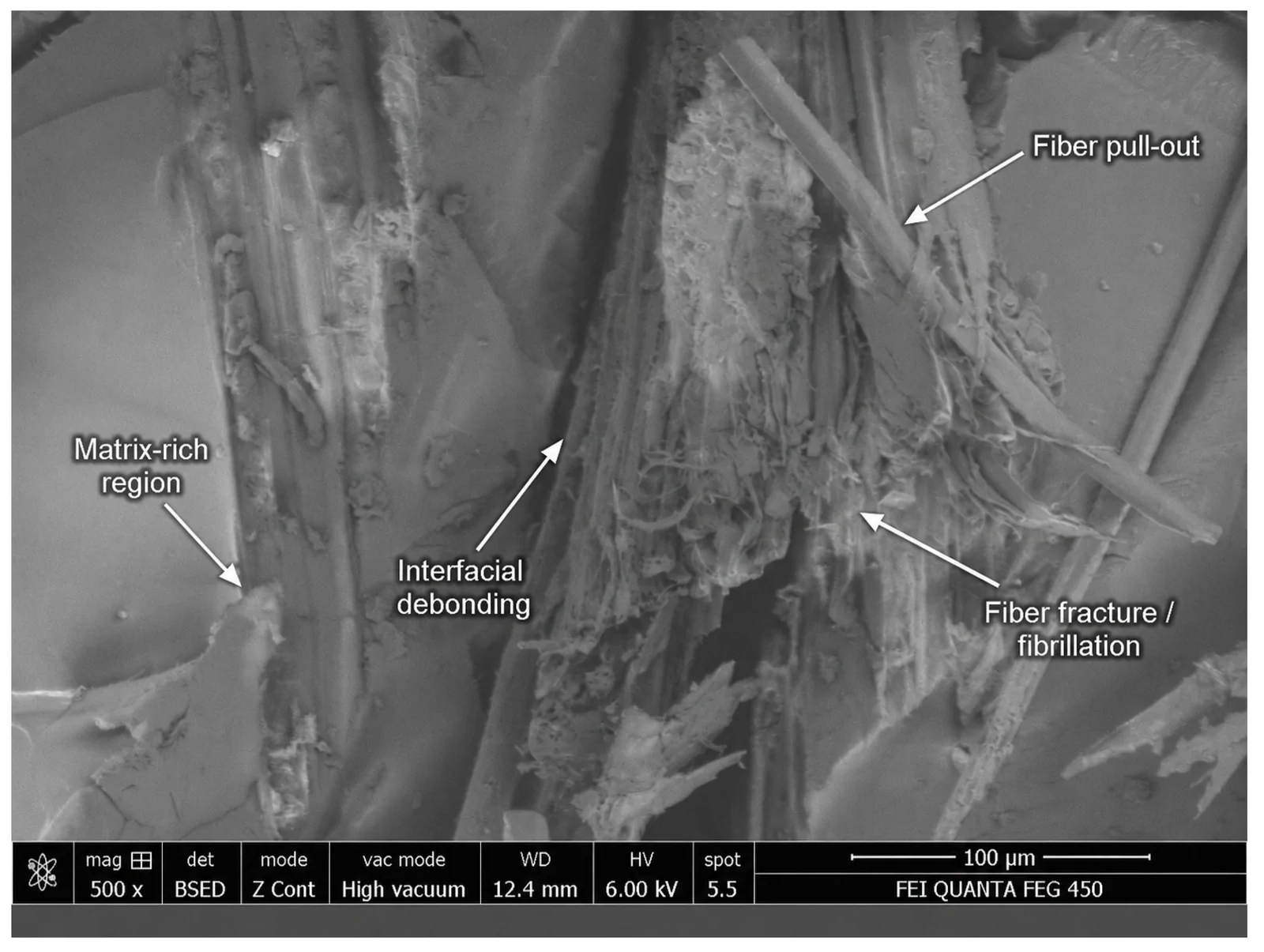
Scanning electron microscopy (SEM) micrograph of sample B containing 30 wt.% false banana fiber and 70 wt.% unsaturated polyester resin (UPR). Arrows indicate representative fractographic features, including matrix-rich regions, interfacial debonding/separation, fiber fracture/fibrillation, and exposed/protruding fibers.

The SEM image of specimen B, containing 30 wt.% false banana fiber and 70 wt.% UPR, is presented in Figure 5.

Figure 5 reveals that sample B had a more irregular fracture surface compared with sample A, with elongated, fractured, and fibrillated false banana fibers visible within the polyester matrix. The principal features marked in Figure 5 include a matrix-rich region, interfacial debonding/separation, fiber fracture/fibrillation, and an exposed/protruding fiber. These observations indicate less uniform fiber–matrix interaction in some regions of sample B.

The fractured and fibrillated fibers, together with exposed/protruding fiber regions and localized interfacial separation, may be associated with debonding during tensile fracture. In addition, matrix-rich regions and apparent interfacial discontinuities indicate non-uniform matrix coverage in some areas of the composite. Since natural fibers are hydrophilic, whereas unsaturated polyester resin is relatively hydrophobic, interfacial incompatibility may contribute to moisture penetration. This qualitative observation is consistent with the water absorption results, in which sample B exhibited the highest water uptake among the investigated composites.

Compared with sample A, sample B showed a less compact fiber arrangement, more evident fiber fracture/fibrillation, exposed fibers, interfacial separation, and a more irregular fracture morphology. These microstructural characteristics may partly contribute to the lower tensile strength and Young’s modulus and the higher water absorption observed for sample B. However, these relationships are interpreted qualitatively because quantitative interfacial, roughness, and porosity measurements were not performed.

**Figure 6:**
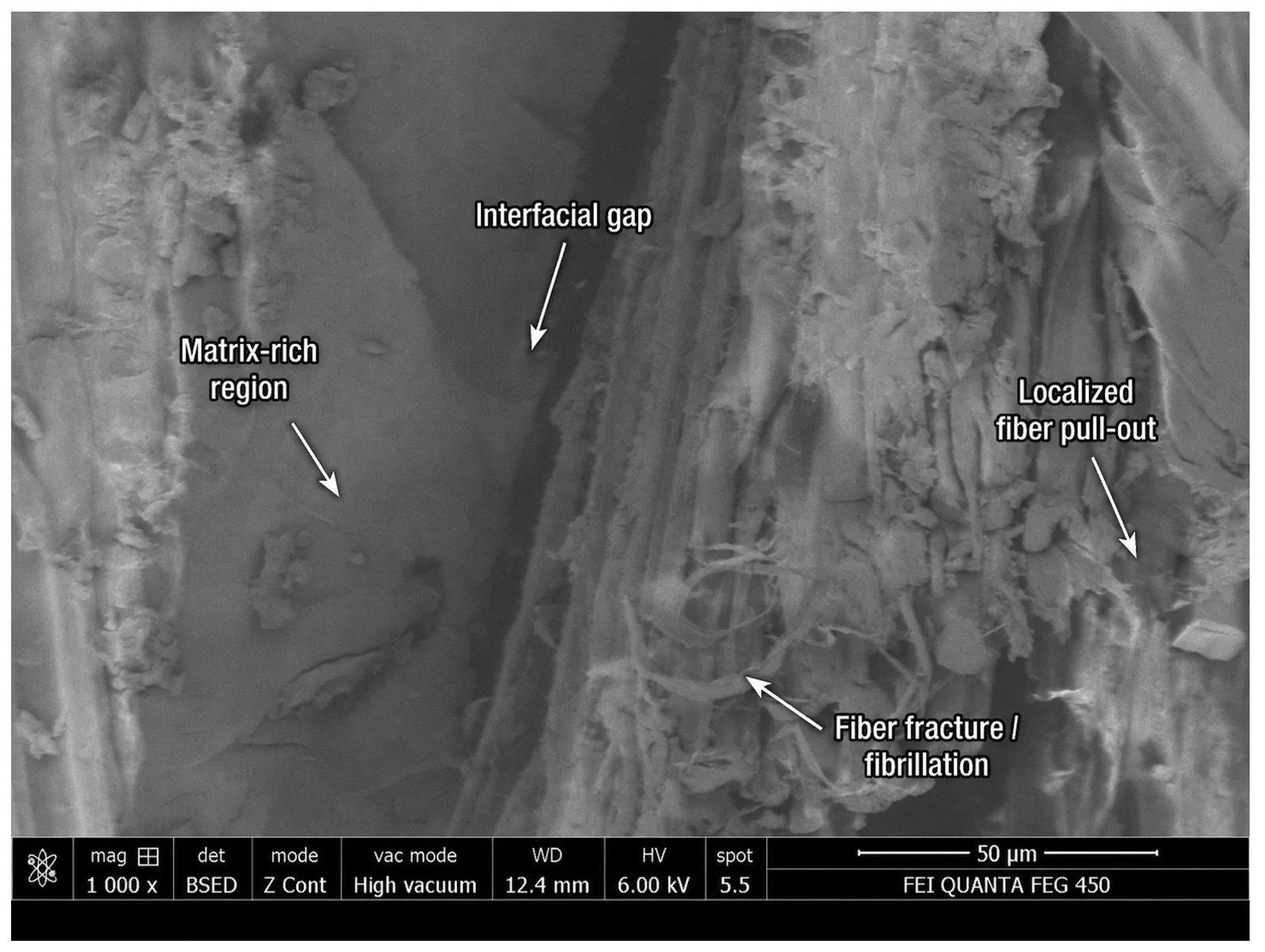
Scanning electron microscopy (SEM) micrograph of sample C containing 15 wt.% jute fiber, 15 wt.% false banana fiber, and 70 wt.% unsaturated polyester resin (UPR). Arrows indicate representative fractographic features, including matrix-rich regions, interfacial separation/gaps, fiber fracture/fibrillation, and exposed fibers.

Figure 6 presents the SEM image of specimen C, containing 15 wt.% jute fiber, 15 wt.% false banana fiber, and 70 wt.% UPR.

Figure 6 reveals the presence of natural fibers embedded within the polyester matrix, together with matrix-rich regions and localized fiber–matrix interfacial discontinuities. The principal fractographic features identified in Figure 6 include matrix-rich regions, interfacial separation/gaps, fiber fracture/fibrillation, and exposed fibers. Compared with sample B, the sample C exhibited a relatively more balanced fracture morphology, with both matrix-covered and fiber-rich regions visible on the fractured surface.

Localized interfacial separation/gaps, exposed fibers, and fiber fracture/fibrillation can also be observed, indicating that fiber–matrix interaction was not completely uniform throughout the composite. However, matrix-covered fiber regions are also present. This intermediate microstructural behavior is consistent with the density, water absorption, and tensile results, for which sample C generally exhibited values between those of samples A and B.

Overall, the SEM observation of sample C indicates that the hybrid composite exhibited a mixed fracture morphology combining features observed in the two single-fiber composite systems. The coexistence of these features suggests that more than one fracture mechanism may have been involved during tensile failure. Overall, the qualitative SEM observations are consistent with the intermediate mechanical and physical behavior of the hybrid composite. Further improvement in fiber–matrix interaction, for example, through fiber surface treatment or processing optimization, may enhance composite performance.

### 3.4. Tensile Properties

**Figure 7:**
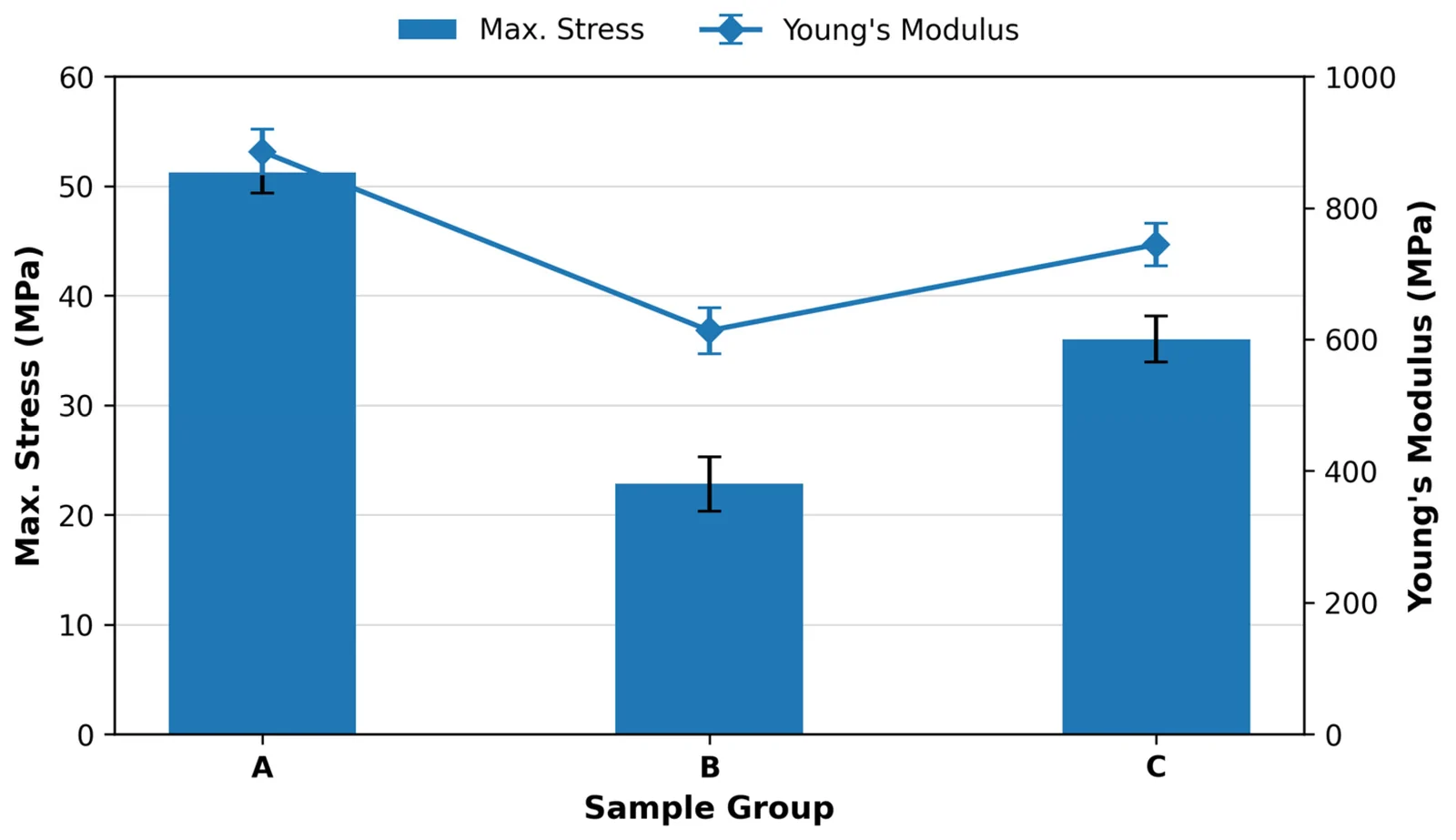
Tensile strength and Young’s modulus of the fiber-reinforced composite specimens.

Table 4 provides a summary of the tensile characteristics of the composite specimens, whereas Figure 7 presents a graphical comparison of the corresponding results.

As shown in Table 4, sample A, which contained only jute fiber reinforcement, showed the best tensile behavior among the composite groups examined. The maximum force, tensile stress, strain, and Young’s modulus recorded for sample A were 4525.0 N, 51.20 MPa, 5.85%, and 885.60 MPa, respectively. These findings show that the jute fiber-reinforced composite had greater load-carrying ability and rigidity compared with the other composites. The enhanced tensile properties may be attributed to improved interfacial adhesion, more efficient load transfer from the UPR matrix to the jute fibers, and the denser fiber configuration evident in the SEM images.

Sample B, reinforced solely with false banana fiber, exhibited the lowest tensile strength and stiffness among the tested composites. The maximum force, tensile stress, strain, and Young’s modulus values obtained for this sample were 2050.0 N, 22.80 MPa, 7.40%, and 613.20 MPa, respectively. Although sample B showed the highest strain value, its lower tensile stress and modulus indicate limited load-bearing capability and reduced rigidity. This behavior may be attributed to weaker fiber–matrix bonding, fiber pull-out, interfacial voids, and nonuniform resin impregnation, as observed in the scanning electron microscopy (SEM) images. The greater strain value suggests that the composite experienced higher deformation before fracture, possibly due to less effective stress transfer and localized debonding at the fiber–matrix interface.

Sample C exhibited moderate tensile properties compared with the single-fiber composites. The maximum force, tensile stress, strain, and Young’s modulus values recorded for sample C were 3235.0 N, 36.00 MPa, 6.40%, and 744.15 MPa, respectively. These values were higher than those of sample B but lower than those of sample A, indicating that hybridization resulted in a balanced mechanical response. Incorporating jute fibers into the hybrid system increased its tensile strength and stiffness relative to the composite reinforced solely with false banana fibers; however, the resulting properties remained below those of the jute-only composite. These findings indicate that the tensile response of the hybrid composite depended on the combined effects of reinforcement type, fiber dispersion, and interfacial adhesion between the fibers and matrix.

Overall, the tensile results indicate that the type of natural fiber strongly influenced the mechanical performance of the unsaturated polyester resin (UPR)-based composites. The sample A exhibited the most favorable tensile properties, whereas the sample B showed the lowest strength and stiffness but the highest deformation capacity. The sample C provided intermediate properties, demonstrating its potential for applications requiring a balance between tensile performance and deformation capability.

The sample C exhibited moderate tensile behavior, indicating that the combined use of jute and false banana fibers may provide a balanced relationship between strength, stiffness, and deformation capacity in natural fiber-reinforced polymer composites.

The tensile behavior obtained in the present work agrees with previous studies reporting that the mechanical characteristics of natural fiber-reinforced polymer composites are strongly influenced by fiber type, fiber morphology, fiber dispersion, and fiber–matrix interfacial bonding [42]. Previous studies on jute fiber-reinforced polymer composites have similarly shown that efficient load transfer across the fiber–matrix interface can improve both tensile strength and stiffness [43]. Accordingly, the higher tensile strength and Young’s modulus observed for sample A in this study may be associated with improved fiber packing, sufficient matrix coverage, and stronger interfacial adhesion.

In contrast, the lower tensile strength of the sample B may be associated with fiber pull-out, interfacial debonding, and less effective stress transfer, as supported by the SEM observations. The intermediate tensile properties of the sample C are also consistent with previous studies indicating that natural fiber hybridization can provide a balanced mechanical response by combining the advantages and limitations of different fiber types [44].

To place the tensile performance of the developed composites in a broader context, the obtained results were compared with the literature data for synthetic fiber-reinforced unsaturated polyester systems. Balakrishnan et al. [45] reported a tensile strength of approximately 440 MPa and a tensile modulus of 28 GPa for a glass fiber-reinforced unsaturated polyester composite, illustrating the substantially higher load-bearing capacity and stiffness achievable with conventional glass fiber reinforcement. Zhang et al. [46] reported a tensile strength of approximately 714.71 MPa for continuous aramid fiber-reinforced unsaturated polyester composites, while surface modification of the aramid fibers further increased the tensile strength to 959.07 MPa. In comparison, the highest tensile strength and Young’s modulus obtained in the present study were 51.20 MPa and 0.886 GPa, respectively, for the jute fiber-reinforced composite. These comparisons demonstrate that glass- and aramid-fiber-reinforced polyester composites can provide substantially higher tensile performance than the natural fiber composites investigated here. However, direct numerical comparison should be made with caution because composite performance is strongly influenced by fiber type and content, reinforcement orientation, interfacial adhesion, fiber surface treatment, and manufacturing method. Therefore, the composites developed in the present study should not be considered direct substitutes for high-performance synthetic fiber composites; rather, their potential lies in the use of renewable lignocellulosic reinforcements, relatively low density, and adequate mechanical performance for lightweight non-load-bearing and semi-structural applications.

## 4. Conclusions

This study comparatively evaluated jute, false banana, and hybrid jute/false banana fiber-reinforced polymer composite (JFBFRPC) fabricated under identical processing conditions at a constant total fiber content of 30 wt.%. The jute fiber-reinforced polymer composite (JFRPC) exhibited the highest tensile strength (51.20 MPa) and Young’s modulus (885.60 MPa), together with the lowest water absorption after 72 h (5.30%). The false banana fiber-reinforced polymer composite (FBFRPC) showed the lowest density (1.16 g/cm^3^) and the highest tensile strain (7.40%), but lower tensile strength and stiffness. The hybrid jute/false banana fiber-reinforced polymer composite (JFBFRPC) exhibited intermediate density, water absorption, tensile strength, Young’s modulus, and strain values, demonstrating a balanced response between the two single-fiber systems.

Qualitative scanning electron microscopy (SEM) fractography revealed clear differences in fiber pull-out, interfacial debonding, matrix coverage, and fracture morphology among the composite groups. Overall, jute/false banana hybridization improved tensile strength, stiffness, and moisture resistance relative to the false banana fiber composite while maintaining a lower density than the jute-only composite. These findings indicate that the hybrid system may be suitable for lightweight non-load-bearing and semi-structural applications where a balance between mechanical performance and density is required.

The present study was limited to physical characterization, tensile testing, and qualitative SEM fractography; thermal, spectroscopic, and crystallographic properties were not experimentally evaluated. Therefore, no conclusions regarding thermal stability, thermal transitions, enthalpy changes, functional groups, or crystallographic structure are drawn from the present results.

Further studies incorporating flexural and impact testing, thermal analyses such as thermogravimetric analysis (TGA) and differential scanning calorimetry (DSC), spectroscopic and crystallographic characterization using Fourier transform infrared spectroscopy (FTIR) and X-ray diffraction (XRD), quantitative microstructural analysis, fatigue testing, long-term durability evaluation, and life-cycle assessment would provide a more comprehensive understanding of the performance and environmental implications of these composites.

## 5. Patents

The work reported in this manuscript has resulted in a registered, examined patent granted by the Turkish Patent and Trademark Office (TURKPATENT). The patent was registered with a 20-year protection period under registration number 2025/021970.

## Figures and Tables

**Table 1:** Weight fractions of the composite formulations.

Sample Group	Jute Fiber (wt.%)	False Banana Fiber (wt.%)	Unsaturated Polyester Resin (wt.%)
A (JFRPC)	30	0	70
B (FBFRPC)	0	30	70
C (JFBFRPC)	15	15	70

**Table 2:** Mean density results of the fiber-reinforced composite specimens.

Sample Group	Density (g/cm^3^)
A	1.22
B	1.16
C	1.19

**Table 3:** Mean water absorption results of the composite specimens.

Sample Group	WA (%)	WA (%)	WA (%)	WA (%)
	2 h	24 h	48 h	72 h
A	1.35	3.20	4.65	5.30
B	1.60	3.85	5.45	6.20
C	1.45	3.50	5.05	5.75

**Table 4:** Tensile test results of the composite specimens.

Sample Group	Max. Force (N)	Max. Stress (MPa)	Max. Stroke (mm)	Strain (%)	Young’s Modulus (MPa)
A	4525.0 ± 165.2	51.20 ± 1.85	2.61 ± 0.72	5.85 ± 1.75	885.60 ± 34.20
B	2050.0 ± 190.0	22.80 ± 2.50	3.10 ± 0.45	7.40 ± 1.10	613.20 ± 35.05
C	3235.0 ± 175.0	36.00 ± 2.10	2.80 ± 0.60	6.40 ± 1.30	744.15 ± 32.53

Where ± = standard deviation.

## Data Availability

The original contributions presented in this study are included in the article. Further inquiries can be directed to the corresponding author.
